# Acute effects of radioiodine therapy on the voice and larynx of basedow-graves patients

**DOI:** 10.1016/S1808-8694(15)31092-2

**Published:** 2015-10-19

**Authors:** Roberta Werlang Isolan-Cury, Osmar Monte, Adriano Namo Cury, Marta Assumpção de Andrada e Silva, André Duprat, Marília Marone, Renata de Almeida, Alexandre Iglesias

**Affiliations:** 1 Master's degree in Health Sciences, Medical Science School, São Paulo Santa Casa (FCMSCSP), Supervisor of the Specialization Course on Voice, Sao Paulo Santa Casa, Professor of the Speech Therapy Course, Medical Science School, São Paulo Santa Casa; 2 Doctoral degree in Endocrinology, FCMSCSP, Head of the Physiology Department and Head of the Endocrinology and Metabology Unit, Sao Paulo Santa Casa; 3 Graduation student in Endocrinology and Metabology, FCMSCSP, Physician, Endocrinologist at the São Paulo Santa Casa; 4 Doctoral thesis in Communication and Semiotics, PUCSP, Adjunct Professor of the Speech Therapy School, FCMSCSP, Adjunct Professor, PUC-SP; 5 Doctoral thesis in Otorhinolaryngology, Adjunct Professor, Otorhinolaryngology Department, São Paulo Santa Casa; 6 Head of the Nuclear Medicine Unit, Nuclimagem - Irmanity of the São Paulo Santa Casa de Misericordia; 7 Otorhinolaryngology specialist, Otorhinolaryngologist; 8 Otorhinolaryngology specialist, Otorhinolaryngologist. Endocrinology and Metabology Unit, Otorhinolaryngology Department, São Paulo Santa Casa de Misericordia

**Keywords:** graves's disease, hyperthyroidism, iodine radioisotopes, voice

## Abstract

Graves's disease is the most common cause of hyperthyroidism. There are three current therapeutic options: anti-thyroid medication, surgery, and radioactive iodine (I 131). There are few data in the literature regarding the effects of radioiodine therapy on the larynx and voice. The aim and the

**Aim:**

os this study was: to assess the effect of radioiodine therapy on the voice of Basedow-Graves patients.

**Material and Method:**

A prospective study was done. Following the diagnosis of Grave's disease, patients underwent investigation of their voice, measurement of maximum phonatory time (/a/) and the s/z ratio, fundamental frequency analysis (Praat software), laringoscopy and (perceptive-auditory) analysis in three different conditions: pre-treatment, 4 days, and 20 days post-radioiodine therapy. Conditions are based on the inflammatory pattern of thyroid tissue (Jones et al. 1999).

**Results:**

No statistically significant differences were found in voice characteristics in these three conditions.

**Conclusion:**

Radioiodine therapy does not affect voice quality.

## INTRODUCTION

Basedow Graves's (GD) Disease may be characterized by the presence of the TSH-receptor autoantibodies (TRAb),^1^^,^^2^ which generate the typical features of diffuse goiter, at times developing ophthalmic disease, and rarely, Graves's dermatopathy.^3^ All of the clinical manifestations of this disease results from the systemic increase in thyroid hormones and their resulting effect on organs and systems.^4^ The main complaints, other than goiter, are the following: anxiety, tremors, tachycardia, weight loss, diarrhea, heat intolerance, fatigue and dyspnea.^3^ The diagnosis is made based on clinical findings, and is confirmed by the presence of TRAb, suppression of TSH, and elevation of free thyroxin (free T4), total thyroxin (total T4) and triiodinethyronin (T3).^1^^,^^3^^,^^4^

There is increased production of carbon dioxide in hyperthyroidism, which activates all of the mechanisms that increase the frequency and depth of breathing.^5^ Increased basal metabolism in hyperthyroid patients leads to a higher consumption of oxygen, vasodilatation and increased blood flow.^6^ In addition to metabolic changes, the pathologically enlarged gland may exert pressure on the trachea and the recurrent laryngeal nerve, which results in dysphagia, respiratory difficulty and noisy breathing.^7^^,^^8^

GD is the most common form of hyperthyroidism. There are currently three approaches to this disease: anti-thyroid medication, surgery in selected cases, and radioactive iodine (I131) therapy.^5^

Anti-thyroid medications are the first line treatment in patients with mild disease, small goiters, children, teenagers, and in special conditions such as pregnancy. I131, on the other hand, is being used increasingly; it is a liquid for oral use and is considered a safe, definitive, inexpensive and easily applied treatment.^9^ Other effects of I131 include early induction of hypothyroidism due to radioactive thyroid tissue destruction. Follicular cells are eliminated by ß radiation, which penetrates from 1 to 2m and destroys the cells that take it up, as well as adjacent cells.^10^^,^^11^ Radiation thyroiditis tends to develop during the first few weeks of treatment; it is evidenced by epithelial edema, follicular derangement and necrosis, and a mononuclear cell infiltrate.^6^ Fibrosis, narrowing of vessels and, eventually, lymphocyte infiltration follow resolution of the acute phase. There have been few reports in the literature about the effects of radioactive iodine on the larynx and, consequently, on voice.

A case was described in 1978 about a woman who received a dose of 7 mCi; one week later she developed hoarseness, which laryngoscopy revealed as being due to right vocal fold paresis. The author stated that paresis might occur as an uncommon complication, and that only one other case had been described (in 1972).^12^ We found a recent paper that described vocal fold paralysis following I131 therapy in a 75-year-old woman who developed hoarseness one week following treatment; indirect laryngoscopy in this patient revealed right vocal fold paralysis. The author raised the hypothesis that vocal fold paralysis was secondary to a mechanical stretching mechanism of the recurrent laryngeal nerve, which itself resulted from focal edema of the adjacent tissues.^13^

The relation between the thyroid and the recurrent laryngeal nerve is well known, especially when voice changes take place following thyroidectomy, due to handling of the nerve.^14^

No study has been published assessing any possible relation between radioactive iodine and the larynx and voice of hyperthyroidic patients. Individuals who use their voice professionally have raised this issue, as well as their concerns about the effect of treatment.

## OBJECTIVE

Given the literature we investigated and the paucity of studies investigating in depth the effects of thyroid dysfunction and radioactive iodine treatment on voice, we decided to assess the acute effects of radioactive iodine-induced laryngeal changes on voice in GD hyperthyroidic patients.

## MATERIAL AND METHOD

This study was approved by the Research Ethics Committee for research on human beings at the institution in which it was undertaken; the protocol number was 023/04.

Forty-five GD hyperthyroidic patients from the Endocrinology and Metabology outpatient unit were interviewed between May 2004 and May 2005, and referred to the Nuclear Medicine unit for radioactive iodine treatment (I131) at a mean dose of 20mCi. Of these 45 patients, 13 were included in this study according to the inclusion/exclusion criteria.

The inclusion criteria were as follows: GD-diagnosed individuals of both sexes, aged between 18 and 50 years, as first referrals to the Endocrinology and Metabology unit for radioactive iodine treatment, not undergoing or not having undergone voice therapy; only patients that came to all of the pre-established evaluation visits were included. The exclusion criteria were as follows: individuals aged over 50 years, menopause, smokers, ex-smokers, and one or more absences from evaluation visits. Patients who presented vocal fold nodules or polyps, Reinke's edema, and cysts and/or grooves were also excluded.

The patients were first contacted two days before the scheduled day for radioactive iodine therapy to measure their interest in the study, for explanations and for clearing doubts. Upon agreement, patients signed a free informed consent form, and were scheduled for assessment.

The procedures took place in three separate moments:

Moment 1. Pre-dose (two hours before ingesting iodine131)

Moment 2. 4–5 days post-dose

Moment 3. 17–18 days post-dose

The procedures included an interview focusing on voice complaints. The next step was a perception of voice analysis, in which the maximum phonation time for the sustained vowel /a/ was measured; the s/z ratio was also calculated. Normal values were 14 seconds for women and 20 second for men;^15^ the normal s/z ratio value is 1.0 second or less.^16^ Voice recording was digital. The fundamental frequency based on the sustained vowel /a/ sample was analyzed using the computer software Praat, version 4.2.31. A standard five seconds were extracted from the mid-portion of the emission, as follows: between the five initial seconds or the final five seconds of the speech sample. Following this selection, zoom was applied to show and analyze the mean fundamental frequency (F0) in the measurement spectrum.

The fundamental frequency for men was defined as 127.61 Hz (standard deviation – 20.26 Hz) and the fundamental frequency for women was defined as 215,42Hz (standard deviation – 53.85 Hz).^17^

Patients underwent videotelelaryngoscopy, in which the following were assessed: presence of aryepiglottic fold edema, the epiglottis, the vocal folds; vocal fold hyperemia; increased vocal fold vascularization, and presence/absence of glottic closure. The TSH and the T4L were measured in all of the moments. The reference ranges for these tests are the following: TSH – 0.45 to 4.50 mIU/mL, and T4L – 0.7 to 1.6 mIU/mL

The voices of patients were presented to three voice specialist speech therapists, who filled in the perception-hearing assessment protocols individually. There was a protocol for each patient, containing sex, age, and sham initials. The GRBASI scale was used for voice analysis. This scale assesses dysphonia (G) globally based on the following features: Roughness (R), Breathiness (B), Asthenia (A), and Strain (S).^16^ In 1996, Instability (I)^18^^,^^19^ was added. For each of these features the following scores may be given: grade 0 (absence), 1 (mild), 2 (moderate) and 3 (severe).

The SPSS (Statistical Package for Social Sciences), version 13.0, was used for the statistical analysis of the sample. The ANOVA test was used for continuous variables and repeated measures. Cochran's Q test was used to analyze vocal fold vascularization, as this is a dichotomic variable. Friedman's test was used for analyzing the ordinal variables edema, vocal fold hyperemia, aryepiglottic fold edema, epiglottic or interarythenoid edema. Bonferroni's test was used for multiple corrections. Significance was achieved at p ≤ 0.05 (5%) for all of the tests. The analysis was descriptive for the variables voice complaints, diagnosis and glottic closure, as these are categoric and of low variability in our sample. Krippendorff's concordance and reliability test was applied to the variables of the perception-hearing analysis done by three speech therapists, who acted as referees. Alpha significance was attained between 0.60 and 0.80, and unconditional alpha concordance was attained between 0.80 and 1.00. If there was no concordance between the referees the majority results were cited and debated.

## RESULTS

**Chart 1:**
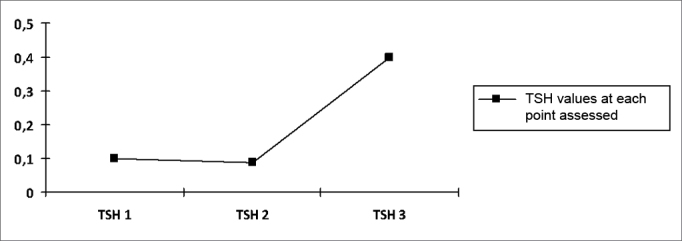
Progression curve showing the mean values of the thyroidstimulating hormone (TSH) in the sample. TSH p=0.493.

**Chart 2:**
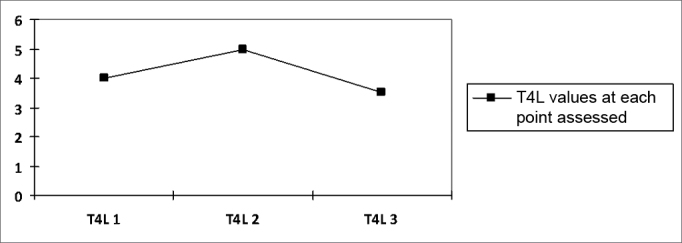
Progression curve showing the mean values of the free thyroxin hormone (T4L) in the sample on the three moments. T4L p=0.518.

There was no statistically significant variation in both thyroid hormone values, which confirms the absence of change from the initial state of hyperthyroidism to the moment 3, as shown on Chart 1, Chart 2.

There were no statistically significant variations for all of the laryngoscopically evaluated parameters (vocal fold edema and hyperemia, aryepiglottic fold edema, epiglottic edema, vascularization and presence/absence of glottic closure), which indicated linearity in these findings and the absence of change in glottic phonation patterns.

Notwithstanding the low rate of agreement between the study referees for the GRBASI scale parameters, there was no statistically significant variation during and throughout the three test moments (Table 1).

**Table 1:** Features of the GRBASI scale at each moment. For the GRBASI scale p>0.05.

	Moment 1	Moment 2	Moment 3
Grade 0	2(15%)	2(15%)	3(23%)
Grade 1	8(61%)	9(70%)	8(61%)
Grade 2	3(23%)	2(15%)	2(15%)
alpha	0,13	0,36	0,11
Roughness 0	2(15%)	4(30%)	4(30%)
Roughness 1	6(46%)	7(53%)	8(61%)
Roughness 2	4(30%)	2(15%)	1(8%)
alpha	0,32	0,41	0,42
Breathiness 0	5(38%)	6(46%)	5(38%)
Breathiness 1	5(38%)	4(30%)	5(38%)
Breathiness 2	3(23%)	3(23%)	3(23%)
alpha	0,72	0,52	0,48
Asthenia 0	6(46%)	11(84%)	11(84%)
Asthenia 1	6(46%)	2(15%)	2(15%)
Asthenia 2	1 (8%)	0	0
alpha	0,44	0,09	0,11
Strain 0	8(61%)	9(70%)	7(53%)
Strain 1	4(30%)	4(30%)	5(38%)
Strain 2	1 (8%)	0	1 (8%)
alpha	0,06	0,07	0,09
Instability 0	8(61%)	10(76%)	8(61%)
Instability 1	5(38%)	3(23%)	5(38%)
Instability 2	0	0	0
alpha	0,17	0,15	0,10

## DISCUSSION

The have been few papers published in the literature about the effects of radioactive iodine therapy on voice quality; existing papers report episodes of vocal fold paresis due to recurrent laryngeal nerve involvement following radioactive iodine therapy.^12^^,^^13^ These studies did not investigate other aspects, such as voice parameters, in the acute post-treatment (radioactive iodine therapy) moments.

The pre-established moments for evaluating the patients were chosen according to the inflammation profile of thyroid tissue in patients undergoing I131 treatment, assessed by direct measurements of cytokines that are usually involved in GD.^20^ The first evaluation took place in moment 1 (pre-dose I131); the earliest point of acute inflammation occurs in moment 2; the evaluation in moment 3 reveals the state of vocal folds and voice quality during the peak inflammation. During these acute inflammatory moments, patients are still hyperthyroidic; there is no mixedema, which could cause laryngeal and voice changes. We included subjects aged 18 or above until age 50 years; these subjects have already gone through voice change but have not yet felt the effects of presbyphonia, which could have masked our results, as applied to voice and laryngeal changes.^15^

Thyroid hormone measurements were made in the three moments to analyze each patient's response to radioactive iodine therapy and to detect possible hormonal variations.

The fact that these patients showed no change in TSH and free T4 levels suggests that hormone variations should not be considered a cause of voice or laryngeal alterations. Hormone alterations develop around 90 days after radioactive iodine therapy,^21^^,^^22^ which was taken into account to eliminate possible interference on results. Phonatory measurements of the vowel /a/ and the s/z ratio yielded no statistically significant variations; descriptive results reveal that most of the patients had low values, probably due to secondary effects to the typical manifestations of hyperthyroidism, such as breathlessness,^13^ fatigue and lack of vocal strength.^23^ Increased glottic muscle performance is needed for theses emissions, which may be lacking in hyperthyroidic patients due to generalized muscle weakness.^24^

According to the clinical experience of the endocrinology unit, the linearity and low frequency of voice-related complaints in our sample may be explained by their relative unimportance compared to the remaining symptoms and discomforts that are typical of hyperthyroidism, except for patients who use their voice professionally; in such cases, voice complaints receive greater attention. In hyperthyroidism there is hear intolerance, increased sweating, mild to extreme weight loss, diarrhea, muscle weakness, nervousness, extreme fatigue and hand tremors.^25^

**Chart 3:**
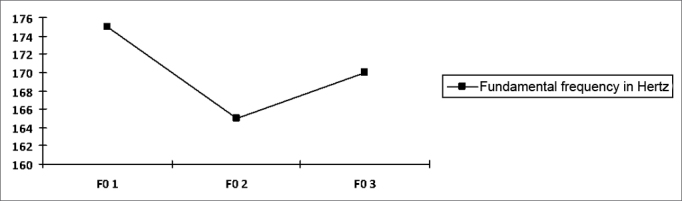
Progression curve for the fundamental frequency (FO) at each moment, shown as the mean in Hertz p=0.400.

There were no statistically significant variations in the computer-analyzed (Praat software) fundamental frequency of voice (measured in Hertz) (Chart 3). This was an expected result, as there was no hormonal variation or progression in the hyperthyroidic state, which would lead to edema and worsening of voice.^27^^,^^28^

Laryngoscopy was aimed at assessing laryngeal mobility and inflammation following I131 use. Findings such as vocal fold edema and hyperemia did not change throughout the three moments; even those cases of mild edema or hyperemia could not be attributed to radioactive iodine therapy. These changes might be related to other diseases, such as laryngeal-pharyngeal reflux. Patients with this condition usually report discomfort in the neck, hoarseness and chronic cough, and may present vocal fold edema.^29^ Diffuse laryngeal hyperemia and interarythenoid edema may be typical findings of laryngeal-pharyngeal reflux patients.^30^

The glottic closure configuration did not change throughout the three evaluation moments (pre- and post-radioactive iodine therapy). Laryngoscopy results revealed that radioactive iodine therapy did not change vocal fold mobility or configuration during the acute inflammation moments that were evaluated; this finding suggests that this type of radiation does not affect the recurrent laryngeal nerve. The statistical concordance test was applied to the results of the perception-auditory analysis, as three referees had analyzed the data independently. Analysis of the GRBASI scale showed no significant variations in all of the parameters (dysphonia grade, roughness, breathiness, asthenia, strain and instability) in the three moments, which is concordant with the absence of hormonal, laryngeal and voice variations.

## CONCLUSION

Our findings suggest that isotope I131 radiation used for treating hyperthyroidic patients due to Graves's Disease does not affect voice quality, and vocal fold mobility and configuration in the acute phase, and does not cause laryngeal inflammation.

## References

[1] Rapoport B, Chazenbalk GD, Jaume JC, McLachlan SM. (1998). The thyrotropin (TSH) receptor: interaction with TSH and autoantibodies. Endocr Rev.

[2] McLachlan SM, Pegg CA, Atherton C, Middleton SL, Clark F (1986). Rees Smith TSH receptor antibody synthesis by thyroid lymphocytes. Clin Endocrinol.

[3] Weetman AP. (2000). Graves' disease. N Engl J Med.

[4] Volpe R. (1988). The immunoregulatory disturbance in autoimmune thyroid disease. Autoimmunity.

[5] Topliss D, Eastman CJ. (2004). 5: Diagnosis and management of hyperthyroidism and hypothyroidism. Med J Aust.

[6] Davies T, Larsen P., Willians RH, Larsen PR (2003).

[7] Cotran RS, Kumar V, Robbins SL, Collins T. (1999). Patologia Estrutural e Funcional.

[8] Velayos JL, Santana HD. (2004). Anatomia da Cabeça e Pescoço.

[9] Thyroid Guidelines Committee. AACE Mecical Guidelines for clinical practice for evaluation and treatment of hyperthyroidism and hypothyroidism Endocr Pract. 2002;8: p. 457 67

[10] Links JM, Wagner HN., Braverman LE, Utiger RD (1991).

[11] Willians ED., Braverman LE, Utiger RD (1991).

[12] Synder S. (1978). Vocal Cord paralysis after radiodine therapy. J Nucl Med.

[13] Coover L. (2000). Permanent iatrogenic vocal cord paralysis after I-131 therapy: a case report and literature review. Clin Nucl Med Sep.

[14] Coelho DH, Boey HP. (2006). Benign parathyroid cyst causing vocal fold paralusis: a case report and review os the literature. Head and Neck.

[15] Behlau M, Madazio G, Feijó D, Pontes D., Behlau M. (2001).

[16] Ferreira L, Pontes P., Ferreira LP. (1990).

[17] Araújo SA, Grellet M, Pereira JC, Rosa MO. (2002). Normatização de medidas acústicas da voz normal. Arq Brás Otorrinolaringol.

[18] Hirano M. (1981).

[19] Dejonckre Remacle, Fresnel Elbaz., Clemente MP. (1996).

[20] Jones B, Kwok C, Kung A. (1999). Effect of radioactive iodine therapy on cytokine production in Graves' disease: transient increases in interleukin-4 (IL-4), IL-6, IL-10, and tumor necrosis factor-alpha, with longer term increases in interferon-gamma production. J Clin Endocrinol Metab Nov.

[21] Aizawa Y, Yoshida K, Kaise N, Fukuzawa H, Kiso Y, Sayama N, Hori H, Abe K. (1997). The development of transient hypothyroidism after iodine-131 treatment patient with Graves' disease: prevalence, mechanism and prognosis. Clin Endocrinol.

[22] Sridama V, Mcormick, Kaplan E, Fauchet R, Degroot L. (1984). Long-term follow-up study of compensated low dose 131 Iodine therapy for Graves' disease. New England J Med.

[23] Gonzáles J. (1992).

[24] Santos K, Vaisman M, Cruz R, Barreto N, Salvador B, Souza A, Nobrega A. (2002). Disfunção muscular esquelética e composição corporal no hipertireoidismo. Arq Bras Endocrinol Metab.

[25] Guyton H. Os Hormônios Metabólicos da Tireóide Tratado de Fisiologia Médica 10^a^ ed. 76 2002 cap.

[27] Bottero S, Minuto I, Modica V, Rispoli G. (1982). Le disfonie nell'hipotiroidismo conclamato. Clin Ter.

[28] Ritter FN. (1967). The effects of hypothyroidism upon the ear, nose and throat. A clinical and experimental study. Laryngoscope.

[29] Cohen JT, Gil Z, Fliss DM. (2005). The reflux symptom index. A clinical tool for the diagnosis of laryngopharyngeal reflux Harefuah.

[30] Ecley CA. (2002). Estudo da Concentração Salivar do fator de crescimento epidérmico em indivíduos com laringite crônica por refluxo laringofaríngeo. Tese de Doutorado - Faculdade de Ciência Médicas da Santa Casa de São Paulo.

